# Racial and Ethnic Disparities in Receipt of ERBB2-Targeted Therapy for Breast Cancer, 2010-2020

**DOI:** 10.1001/jamanetworkopen.2025.8086

**Published:** 2025-05-01

**Authors:** Sudarshan Krishnamurthy, Shelley A. Jazowski, Mya L. Roberson, Katherine Reeder-Hayes, Jasmyn J. Tang, Stacie B. Dusetzina, Utibe R. Essien

**Affiliations:** 1 Department of Internal Medicine, Wake Forest University School of Medicine, Winston-Salem, North Carolina; 2 Department of Health Policy, Vanderbilt University School of Medicine, Nashville, Tennessee; 3 Department of Social Sciences and Health Policy, Wake Forest University School of Medicine, Winston-Salem, North Carolina; 4 Department of Health Policy and Management, Gillings School of Global Public Health, University of North Carolina at Chapel Hill; 5 Division of Oncology, Department of Medicine, UNC School of Medicine, Chapel Hill, North Carolina; 6 Division of General Internal Medicine and Health Services Research, David Geffen School of Medicine, University of California, Los Angeles; 7 Center for the Study of Healthcare Innovation, Implementation and Policy, Greater Los Angeles VA Healthcare System, Los Angeles, California; 8 Associate Editor, *JAMA Network Open*

## Abstract

**Importance:**

Among older women (aged ≥50 years) with ERBB2 (formerly HER2 or HER2/neu)–positive breast cancer, research has shown racial and ethnic disparities in access to ERBB2-targeted therapies, with Black women receiving treatment at lower rates than their White counterparts.

**Objective:**

To examine racial and ethnic disparities in receipt of ERBB2-targeted therapies and changes in receipt over time.

**Design, Setting, and Participants:**

This retrospective cohort study used Surveillance, Epidemiology, and End Results–Medicare linked data from January 1, 2010, to December 31, 2020. Beneficiaries who were diagnosed with ERBB2-positive breast cancer between 2010 and 2019, were aged 66 years or older at diagnosis, were continuously enrolled in Medicare Parts A and B in the 12 months before and after diagnosis, and had localized or regional stage disease at diagnosis were included. Data were analyzed from February through September 2024.

**Exposure:**

Race and ethnicity defined as non-Hispanic Black or African American, Hispanic, or non-Hispanic White.

**Main Outcome and Measures:**

The primary outcome was receipt of ERBB2-targeted therapies in the 12 months after diagnosis of ERBB2-positive breast cancer. Modified Poisson regression was used to evaluate the probability of receiving ERBB2-targeted therapy by race and ethnicity.

**Results:**

Among 12 765 beneficiaries with ERBB2-positive breast cancer (median [IQR] age, 74 [69-80] years; 99.2% female), 8.1% were of Black, 6.9% Hispanic, and 85.0% White race and ethnicity, and 54.2% received ERBB2-targeted therapy. The overall proportion who received ERBB2-targeted therapies increased from 41.3% in 2010-2011 to 64.3% in 2018-2019. Compared with White patients, Black patients had a lower likelihood of receiving ERBB2-targeted therapies in 2010-2011 (adjusted risk ratio [ARR], 0.81; 95% confidence limit [CL], 0.68-0.97), as did Hispanic patients (ARR, 0.75; 95% CL, 0.62-0.92). Racial and ethnic disparities in receipt of ERBB2-targeted therapies narrowed over time, with no significant differences observed across racial and ethnic groups in 2018-2019 for Black patients (ARR, 0.97; 95% CL, 0.87-1.08) and Hispanic patients (ARR, 1.05; 95% CL, 0.95-1.16).

**Conclusions and Relevance:**

These findings suggest a narrowing of racial and ethnic disparities in receipt of ERBB2-targeted therapies over time among older Medicare beneficiaries with ERBB2-positive breast cancer. Future research is needed to understand the practices that contributed to the narrowing of racial and ethnic disparities and to develop implementation strategies to effectively improve the quality and equity of breast cancer care.

## Key Points

**Question:**

Among Medicare beneficiaries diagnosed with ERBB2 (formerly HER2 or HER2/neu)–positive breast cancer between 2010 and 2019, are racial and ethnic disparities associated with receipt of ERBB2-targeted therapies, and do these trends change over time?

**Findings:**

In this cohort study of 12 765 beneficiaries with ERBB2-positive breast cancer, a narrowing of racial and ethnic disparities in receipt of ERBB2-targeted therapies over time was observed.

**Meaning:**

These findings suggest that identifying barriers associated with receipt of ERBB2-targeted therapies, even as guidelines broadly recommend using these drugs, is crucial for improving the quality and equity of breast cancer care.

## Teaser

This cohort study examines racial and ethnic disparities in the receipt of ERBB2 (formerly HER2 or HER2/neu)–targeted therapies and changes in receipt over time among Medicare beneficiaries with ERBB2-positive breast cancer.

## Introduction

Breast cancer is the most common type of cancer among women in the US, with nearly 300 000 estimated new cases in 2022.^1^ Although breast cancer incidence is similar among Black and White women,^2^ breast cancer mortality is particularly high among Black women,^3^ who experience an age-adjusted mortality that is 40% higher than non-Hispanic White women.^4^ Moreover, Black and Hispanic women are less likely to receive guideline-concordant breast cancer therapy,^5^ which may contribute to disparate clinical outcomes and mortality.^6,7,8^

Approximately 15% to 25% of breast cancers are classified as ERBB2 (formerly HER2 or HER2/neu) positive, a classification of breast cancer that is known to be associated with aggressive tumor behavior.^9^ The use of ERBB2-targeted therapies, such as trastuzumab, have dramatically transformed the treatment paradigm and improved clinical outcomes, including disease-free and overall survival.^10,11^ Since 1998, with US Food and Drug Administration (FDA) approval, trastuzumab has been the standard of care for patients with ERBB2-positive breast cancer, with the approval of additional ERBB2-targeted therapies since then. Despite the effectiveness of these therapies, previous studies have shown racial and ethnic disparities in access to ERBB2-targeted therapies, with Black women receiving treatment at lower rates than their White counterparts.^12^ However, these data are older, with limited examination of racial and ethnic disparities in receipt of ERBB2-targeted therapies after 2011, and receipt of newer ERBB2-targeted therapies other than trastuzumab has not been assessed. Furthermore, no studies to our knowledge have examined trends in receipt of these therapies over time.

Although extensive disparities in receipt of novel cancer therapies among historically underserved groups have been observed,^13,14^ there is variability in whether these trends improve over time or whether disparities in medication access persist. Striving to achieve pharmacoequity, or the goal of ensuring that all patients have equitable access to treatment, in ERBB2-positive breast cancer must be a priority.^15^ Furthermore, it is crucial to elucidate whether existing disparities may be associated with delayed adoption or long-term access gaps among racially and ethnically minoritized groups.

The objectives of this study were to examine the receipt and changes in use of ERBB2-targeted therapies by race and ethnicity over time among older adults with localized or regional stage ERBB2-positive breast cancer. We hypothesized that patients from racially and ethnically minoritized groups would have persistently lower receipt of ERBB2-targeted therapies for the treatment of breast cancer.

## Methods

### Data Source and Study Cohort

**Figure 1.**
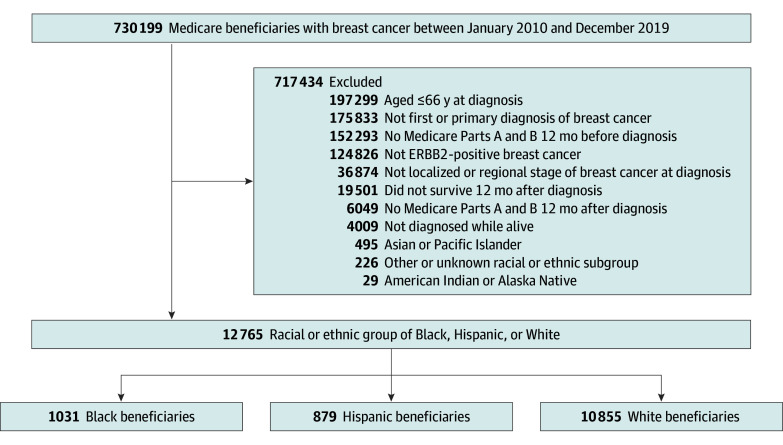
Flow Diagram for the Study Cohort

In this cohort study, we used the Surveillance, Epidemiology, and End Results (SEER)–Medicare linked database to identify fee-for-service beneficiaries diagnosed with primary ERBB2-positive breast cancer between January 1, 2010, and December 31, 2019 (Figure 1). This study was reviewed by the Vanderbilt University Medical Center Institutional Review Board. A waiver of informed consent was provided as the research could not be practicably conducted without the requested waiver. The study followed the Strengthening the Reporting of Observational Studies in Epidemiology (STROBE) reporting guideline.

ERBB2 status, which was not available in the SEER registries prior to 2010, was defined using the breast subtype variable (specifically, individuals with ERBB2-positive/hormone receptor–positive and ERBB2-positive/hormone receptor–negative disease). Eligible patients were aged 66 years or older at diagnosis, survived for at least 12 months after diagnosis, were continuously enrolled in Medicare Parts A and B in the 12 months before and after diagnosis, and had localized or regional stage disease at diagnosis. We excluded beneficiaries who were diagnosed at death or autopsy or were enrolled in health maintenance organization plans (due to incomplete ascertainment of claims).^12^

### Study Outcome

The primary outcome was receipt of any clinician-administered therapy indicated to treat ERBB2-positive breast cancer that was available for adjuvant use in the 12 months after diagnosis (herein referred to as ERBB2-targeted therapies). Specifically, we included trastuzumab- and pertuzumab-based products, their combinations, and ado-trastuzumab emtansine. We used Healthcare Common Procedure Classification System codes to identify at least 1 Medicare Part B claim for a brand name or biosimilar ERBB2-targeted therapy (eTable 1 in Supplement 1).

### Independent Variables and Covariates

The key independent variables were race and ethnicity, which were conceptualized as both sociopolitical and cultural constructs and operationalized to reflect the influence of forces that lead to institutional inequity and interpersonal discrimination.^16,17^ We defined race and ethnicity using the Research Triangle Institute variable, which is based on an algorithm that used name (from the US census) and state of residence to improve the identification of Hispanic and Asian or Pacific Islander Medicare beneficiaries.^18,19,20^ Our analysis focused on the following mutually exclusive categories: non-Hispanic Black or African American (hereafter referred to as Black), Hispanic, and non-Hispanic White (hereafter referred to as White). Due to limited sample sizes, we were unable to assess all racial and ethnic groups and, thus, excluded 29 American Indian or Alaska Native beneficiaries and 495 Asian or Pacific Islander beneficiaries from the analysis.

Covariates included age at diagnosis, sex, census region (based on the location of each SEER registry), year of diagnosis, summary stage at diagnosis (localized [cancer that is limited to the place of origin] or regional [cancer that has spread to the nearby lymph nodes, tissues, or organs]), hormone receptor status (positive or negative), medical comorbidities (measured in the 12 months prior to diagnosis using the Klabunde modification of the Charlson Comorbidity Index and including diagnoses of acute and history of myocardial infarction, congestive heart failure, peripheral vascular disease, cerebrovascular disease, chronic obstructive pulmonary disease, dementia, hemiplegia or paraplegia, diabetes, moderate to severe kidney disease, mild liver disease, moderate to severe liver disease, peptic ulcer disease, rheumatologic disease, and AIDS),^21^ and Medicare Part D Low-Income Subsidy status (full or partial or no subsidies).

### Statistical Analysis

We compared demographic and clinical characteristics across racial and ethnic groups using χ^2^ tests. We used modified Poisson regression with robust error variance to assess the association between race and ethnicity and receipt of ERBB2-targeted therapies.^22^ We estimated the probability of receiving treatment during the study period (2010-2020) and in 2-year increments (based on year of diagnosis 2010-2011, 2012-2013, etc). Consistent with the National Academy of Medicine’s definition of racial and ethnic disparities,^23,24^ models were adjusted for beneficiaries’ health status (age at diagnosis, summary stage at diagnosis, hormone receptor status, and medical comorbidities) and geographic region.^23,24,25^

We also conducted several sensitivity analyses to assess the robustness of our findings. First, since prior research on breast cancer disparities has predominantly focused on female patients,^6,12,24,25^ we restricted the cohort to women diagnosed with ERBB2-positive breast cancer. Second, clinical practice and treatment guidelines are often based on the TNM staging system^26,27^; therefore, we restricted the cohort to beneficiaries diagnosed with stage I to III disease (defined using derived American Joint Commission on Cancer, combined summary, and derived extent of disease staging variables). Finally, we adjusted our models for a marker of socioeconomic status, the Medicare Part D Low-Income Subsidy status, to determine whether observed racial and ethnic differences in receiving ERBB2-targeted therapies were attenuated.^24,25^ All analyses were conducted from February through September 2024 using SAS Studio, version 9.4 (SAS Institute Inc), and statistical tests with a 2-sided *P* < .05 were considered statistically significant.

## Results

### Cohort Characteristics

Among 12 765 Medicare beneficiaries diagnosed with ERBB2-positive breast cancer between 2010 and 2019 (median [IQR] age at diagnosis, 74 [69-80] years; 99.2% female and 0.8% male), 8.1% were of Black, 6.9% Hispanic, and 85.0% White race and ethnicity (Table 1). Overall, 54.2% received ERBB2-targeted therapies in the 12 months after diagnosis. eTable 2 in Supplement 1 lists the characteristics of treated vs untreated individuals. Most beneficiaries were diagnosed with localized stage cancer (65.5%), had hormone receptor–positive disease (73.3%), and did not have other medical comorbidities prior to diagnosis (58.7%). Aside from sex and year of diagnosis, all demographic and clinical characteristics were significantly different across Black, Hispanic, and White beneficiaries (Table 1).

**Table 1.** Cohort Characteristics of Medicare Beneficiaries With ERBB2 (Formerly HER2 or HER2/Neu)–Positive Breast Cancer, by Race and Ethnicity

Characteristic	Study Population (N = 12 765)^a^	Black (n = 1031)^a^^,^^b^	Hispanic (n = 879)^a^^,^^b^	White (n = 10 855)^a^^,^^b^	*P* value
Age at diagnosis, y					
≤69	3371 (26.4)	315 (30.6)	252 (28.7)	2804 (25.8)	<.001
70-73	2913 (22.8)	236 (22.9)	225 (25.0)	2452 (22.6)	
74-79	3213 (25.2)	245 (23.8)	211 (24.0)	2757 (25.4)	
≥80	3268 (25.6)	235 (22.8)	191 (21.7)	2842 (26.2)	
Low-income subsidy^c^					
Full or partial	1948 (15.3)	351 (34.0)	417 (47.4)	1180 (10.9)	<.001
None	10 817 (84.7)	680 (66.0)	462 (52.6)	9675 (89.1)	
Census region^d^					
Northeast	4034 (31.6)	319 (30.9)	183 (20.8)	3532 (32.5)	<.001
Midwest	941 (7.4)	88 (8.5)	12 (1.4)	841 (7.8)	
South	4411 (34.6)	490 (47.5)	313 (35.6)	3608 (33.2)	
West	3379 (26.5)	134 (13.0)	371 (42.2)	2874 (26.5)	
Year of diagnosis					
2010	1215 (9.5)	106 (10.3)	70 (8.0)	1039 (9.6)	.43
2011	1213 (9.5)	102 (9.9)	87 (9.9)	1024 (9.4)	
2012	1247 (9.8)	93 (9.0)	104 (11.8)	1050 (9.7)	
2013	1223 (9.6)	103 (10.0)	87 (9.9)	1033 (9.5)	
2014	1346 (10.5)	93 (9.0)	93 (9.0)	1160 (10.7)	
2015	1451 (11.4)	117 (11.4)	88 (10.0)	1246 (11.5)	
2016	1400 (11.0)	114 (11.1)	102 (11.6)	1184 (10.9)	
2017	1329 (10.4)	123 (11.9)	83 (9.4)	1123 (10.4)	
2018	1216 (9.5)	85 (8.2)	91 (10.4)	1040 (9.6)	
2019	1125 (8.8)	95 (9.2)	74 (8.4)	956 (8.8)	
Summary stage at diagnosis^e^					
Local	8364 (65.5)	598 (58.0)	558 (63.5)	7208 (66.4)	<.001
Regional	4401 (34.5)	433 (42.0)	321 (36.5)	3647 (33.6)	
Hormone receptor status					
Positive	9355 (73.3)	701 (68.0)	633 (72.0)	8021 (73.9)	<.001
Negative	3410 (26.7)	330 (32.0)	246 (28.0)	2834 (26.1)	
Comorbidities^f^					
0	7490 (58.7)	478 (46.4)	514 (58.5)	6498 (59.9)	<.001
1	2631 (20.6)	229 (22.2)	158 (18.0)	2244 (20.7)	
≥2	2644 (20.7)	324 (31.4)	207 (23.6)	2113 (19.5)	
Treated within 12 mo of diagnosis					
Yes	6916 (54.2)	529 (51.3)	444 (50.5)	5943 (54.8)	.008
No	5849 (45.8)	502 (48.7)	435 (49.5)	4912 (45.3)	

^a^ Less than 1% of the population was male.

^b^ Race and ethnicity were defined using the Research Triangle Institute variable.

^c^ Receipt of the Medicare Part D Low-Income Subsidy was measured at diagnosis.

^d^ Census regions were based on beneficiaries’ Surveillance, Epidemiology, and End Results registry (eg, Massachusetts was categorized as Northeast).

^e^ Localized stage describes cancer that is limited to the place where it started, with no signs of spread. Regional stage describes cancer that has spread to the nearby lymph nodes, tissues, or organs.^27^

^f^ Comorbidities were measured in the 12 months before diagnosis using the Klabunde modification of the Charlson Comorbidity Index. Comorbidities captured in this score include diagnoses of acute and history of myocardial infarction, congestive heart failure, peripheral vascular disease, cerebrovascular disease, chronic obstructive pulmonary disease, dementia, hemiplegia or paraplegia, diabetes, moderate to severe kidney disease, mild liver disease, moderate to severe liver disease, peptic ulcer disease, rheumatologic disease, and AIDS.

### Receipt of ERBB2-Targeted Therapies by Race and Ethnicity

We observed low rates of ERBB2-targeted therapy use across racial and ethnic groups over the study period, although use increased over time. The overall proportion who received ERBB2-targeted therapies increased from 41.3% in 2010-2011 to 64.3% in 2018-2019. From 2010 to 2020, 54.7% of White patients received ERBB2-targeted therapy, whereas 51.3% and 50.5% of Black and Hispanic patients, respectively, received ERBB2-targeted therapy (Table 2). Compared with White beneficiaries, Black beneficiaries (adjusted risk ratio [ARR], 0.93; 95% confidence limit [CL], 0.87-0.98) and Hispanic beneficiaries (ARR, 0.88; 95% CL, 0.82-0.93) had a lower likelihood of receiving ERBB2-targeted therapy during the study period (Table 2).

**Table 2.** Receipt of ERBB2 (Formerly HER2 or HER2/Neu)−Targeted Therapies Among Medicare Beneficiaries With ERBB2-Positive Breast Cancer, by Race and Ethnicity

Race and ethnicity	Rate of therapy initiation, %	RR (95% CL)^a^	
		Unadjusted	Adjusted
Black	51.3	0.94 (0.88-1.00)	0.93 (0.87-0.98)
Hispanic	50.5	0.93 (0.86-0.99)	0.88 (0.82-0.93)
White	54.7	1 [Reference]	1 [Reference]

Abbreviations: CL, confidence limit; RR, risk ratio.

^a^ Fully modified Poisson regression results are displayed in eTable 4 in Supplement 1.

**Figure 2.**
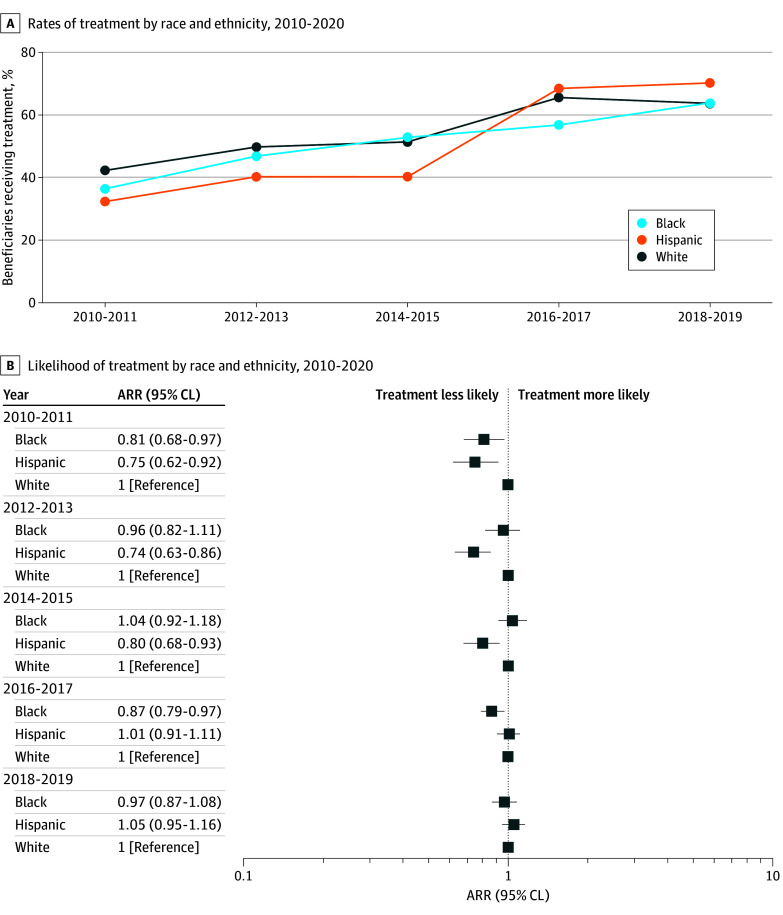
Receipt of ERBB2 (Formerly HER2 or HER2/Neu)–Targeted Therapies Over Time by Race and Ethnicity A, Time intervals were based on year of diagnosis, and receipt of ERBB2-targeted therapies was measured in the 12 months after diagnosis. B, Adjusted risk ratios (ARRs) were estimated using modified Poisson regression models that adjusted for health status and geographic region. CL indicates confidence limit.

From 2010-2011 to 2018-2019, receipt of ERBB2-targeted therapies increased from 36.5% to 63.9% for Black beneficiaries, from 32.5% to 70.3% for Hispanic beneficiaries, and from 42.5% to 63.9% for White beneficiaries (Figure 2A). In adjusted models, Black beneficiaries (ARR, 0.81; 95% CL, 0.68-0.97) and Hispanic beneficiaries (ARR, 0.75; 95% CL, 0.62-0.92) were less likely than their White counterparts to receive treatment in 2010-2011 (Figure 2B). By 2018-2019, racial and ethnic differences in receipt of ERBB2-targeted therapies were no longer statistically significant for Black beneficiaries (ARR, 0.97; 95% CL, 0.87-1.08) and Hispanic beneficiaries (ARR, 1.05; 95% CL, 0.95-1.16).

### Trends in the Receipt of Specific ERBB2-Targeted Therapies

Over time, the use of various ERBB2-targeted therapies shifted among all beneficiaries, from 100% of patients receiving trastuzumab in 2010-2011 to 50.0% and 45.5% of patients receiving trastuzumab or combination therapy (eg, trastuzumab plus pertuzumab), respectively, in 2018-2019 (eTable 3 in Supplement 1). Receipt of ERBB2-targeted therapies also varied by race and ethnicity, with 100% of patients from all racial and ethnic groups receiving trastuzumab in 2010-2011; however, 48.7% of Black patients, 46.6% of Hispanic patients, and 50.4% of White patients received trastuzumab in 2018-2019 (eTable 3 in Supplement 1).

### Covariates Associated With Receipt of ERBB2-Targeted Therapies

In secondary analyses, we examined the demographic and clinical factors associated with the receipt of ERBB2-targeted therapies. Compared with beneficiaries aged 69 years or younger, those aged 80 years or older were less likely to ever receive therapy (ARR, 0.46; 95% CL, 0.44-0.49) (eTable 4 in Supplement 1). The low likelihood of treatment use among beneficiaries aged 80 years or older persisted from 2010-2011 (ARR, 0.42; 95% CL, 0.36-0.49) (eTable 5 in Supplement 1) to 2018-2019 (ARR, 0.52, 95% CL, 0.47-0.58) (eTable 5 in Supplement 1). Residents of the South had a lower probability of ever receiving treatment compared with those residing in the Northeast (ARR, 0.70; 95% CL, 0.67-0.73) (eTable 4 in Supplement 1); however, this regional variation in the receipt of ERBB2-targeted therapies narrowed from 2010-2011 (ARR, 0.47; 95% CL, 0.41-0.54) to 2016-2017 (ARR, 0.96; 95% CL, 0.90-1.03) (eTable 5 in Supplement 1).

### Sensitivity Analyses

In analyses that restricted the cohort to female beneficiaries, assessed beneficiaries diagnosed with stage I to III cancer, and adjusted for socioeconomic status, findings were consistent with our primary analysis. These findings are shown in eTables 6 and 7 in Supplement 1.

## Discussion

In this cohort study of older Medicare beneficiaries diagnosed with ERBB2-positive breast cancer between 2010 and 2019, we observed a narrowing of racial and ethnic disparities in the receipt of ERBB2-targeted therapies over time. Specifically, compared with White beneficiaries, the difference in the rates of receipt decreased from 6% to 0% for Black beneficiaries and from 10% to −6% for Hispanic beneficiaries. The overall proportion of the population receiving ERBB2-targeted therapies increased from 41.3% in 2010-2011 to 64.3% in 2018-2019. Although use of ERBB2-targeted therapy has increased over time, the large percentage of beneficiaries who are not receiving guideline-recommended care remains a concern.^28^

Prior research that investigated racial and ethnic disparities in trastuzumab receipt among Medicare beneficiaries with ERBB2-positive breast cancer has shown mixed results. Two studies reported that Black women were less likely than White women to receive trastuzumab,^12,29^ whereas 1 study did not observe any significant differences in ERBB2-targeted therapy initiation between Black and White beneficiaries.^30^ Aside from research focused solely on the Medicare population, a 2005-2008 study using the National Comprehensive Cancer Network Breast Cancer Outcomes Database found no significant differences in ERBB2-targeted therapy receipt by race and ethnicity.^31^ Our findings expand the literature by assessing racial and ethnic disparities in the receipt of ERBB2-targeted therapies over time and providing new evidence of narrowing disparities as the FDA approval and availability of these therapies increased.

Despite well-documented racial and ethnic disparities in breast cancer treatment,^6,12,32^ our findings show a narrowing of disparities in the receipt of ERBB2-targeted therapies over time among Medicare beneficiaries with ERBB2-positive breast cancer. It is possible that this narrowing of disparities may have been largely driven by the gaps in receipt of ERBB2-targeted therapies that were successfully bridged in the US South. The reversal of a disparity between Hispanic and White patients (although not statistically significant) is also noteworthy and requires further study. More research is needed to identify the strategies and practices that contributed to the narrowing of disparities in the receipt of ERBB2-targeted therapy, as such strategies may be implemented to improve the quality and equity of breast cancer care more broadly. Additionally, with FDA approval and availability of several new ERBB2-targeted therapies and biosimilars during the study period, future research is needed to determine whether access to these newer therapies may have contributed to the narrowing of observed racial and ethnic disparities. Nonetheless, although these therapies possess a high level of efficacy in ERBB2-positive breast cancer,^33^ we observed low overall use as recent as 2020 and a slow increase in uptake over time among all Medicare beneficiaries.

There are several mechanisms that may explain low uptake of ERBB2-targeted therapies in our study, even in the later periods of our analysis. One factor that remained associated with a low likelihood of receiving ERBB2-targeted therapies across all periods was age at diagnosis. Beneficiaries aged 80 years or older were consistently less likely to receive ERBB2-targeted therapies than those aged 69 years or younger. Other potential factors that may have influenced ERBB2-targeted therapy use include clinicians’ implicit bias, as seen with treatments for other illnesses^34^; clinicians’ reluctance to prescribe therapy to patients with cardiac risk factors and/or comorbidities^35^; and patients’ preferences for treatment.^36^ In addition, structural factors, such as insurance coverage and cost-sharing,^37,38^ reliable transportation,^39^ a dearth of medical oncologists resulting in a lack of access to care,^6^ and work or family responsibilities,^40^ have also been associated with decreased treatment initiation. Finally, other social determinants of health, such as health literacy and mistrust of the health care system, also may have been associated with certain individuals’ engagement in their care and limited treatment options.^40,41^

ERBB2-targeted therapies are the standard of care in clinical practice guidelines for the treatment of ERBB2-positive breast cancer.^42^ Previous studies have suggested that treatment delays of 3 to 6 months are associated with worse survival in individuals with breast cancer.^43,44^ Thus, the years-long disparities between Black and White and Hispanic and White patients in ERBB2 therapy initiation between 2010-2011 and 2018-2019 may have resulted in poor clinical outcomes, including a widening of the mortality gap, among racially and ethnically minoritized populations with breast cancer. Improving early access to high-quality, evidence-based therapies is crucial to advancing health outcomes for all individuals with breast cancer.^45^ Further study of barriers to accessing ERBB2-targeted therapies is needed to target interventions that address determinants at various levels, including health and social policy, research infrastructure, clinician, and patient,^45^ and ensure that implementation of guideline-recommended breast cancer therapy is equitable from its initial availability. Furthermore, research that identifies pharmacoequity strategies and best practices that contributed to the narrowing of racial and ethnic disparities may help inform policy changes in areas where disparities still persist. These practices may also guide the improvement of equitable access for other breast cancer therapies and populations beyond those with ERBB2-positive cancers.

### Limitations

This study has several limitations. First, our analysis focused on fee-for-service Medicare beneficiaries; thus, the findings may not generalize to individuals with other forms of public or private insurance coverage, those who are uninsured, or those younger than 65 years. Second, although the Research Triangle Institute variable was based on an algorithm that improves the identification of certain racial and ethnic groups, we were unable to assess treatment use among American Indian or Alaska Native and Asian or Pacific Islander beneficiaries due to small sample sizes. Third, we were unable to assess the reasons for not receiving ERBB2-targeted therapy (eg, patient or clinician preferences, structural barriers), which are crucial for determining interventions aimed at improving treatment initiation. Fourth, SEER registries only capture stage at diagnosis; thus, we were unable to analyze beneficiaries with recurrent metastatic cancer. As such, future research is needed to understand the uptake of and disparities in the receipt of ERBB2-targeted therapies in this population. Fifth, our analysis focused on the receipt of clinician-administered therapies indicated to treat ERBB2-positive breast cancer; therefore, future studies are needed to understand disparities and trends in the use of orally administered medications. Sixth, although we used a common index to identify comorbidities, this measure may not reflect the specific clinical conditions (eg, cardiac risk factors) that may influence breast cancer treatment decisions. Finally, given the limited availability of such data, our analysis did not account for certain barriers to health equity (eg, structural racism, implicit bias) for which further study is needed.

## Conclusions

In this cohort study of older Medicare beneficiaries with ERBB2-positive breast cancer, we observed a narrowing of racial and ethnic disparities in ERBB2-targeted therapy from 2010-2011 to 2018-2019. Though these findings are promising, future research is needed to understand how improved equitable access to ERBB2-targeted therapy is associated with disparities in breast cancer outcomes. Furthermore, identifying structural barriers and prescriber-specific factors associated with the overall likelihood of receiving ERBB2-targeted therapy in more recent time frames, even as guidelines broadly recommend use of these drugs, is crucial to improving quality of breast cancer care. Interventions that target drivers of noninitiation, particularly among racially and ethnically minoritized groups and older patients, may help ensure pharmacoequity for individuals diagnosed with ERBB2-positive breast cancer.

## Notes

Supplement 1. **eTable 1. **ERBB2-Targeted Therapies Included in Analysis **eTable 2. **Cohort Characteristics by Treatment Status **eTable 3. **Receipt of Various ERBB2-Targeted Therapies Among Medicare Beneficiaries With ERBB2-Positive Breast Cancer, by Race and Ethnicity **eTable 4. **Results of Multivariable Model Through the Duration of the Study Period **eTable 5. **Results of Multivariable Time-Specific Models **eTable 6.** Sensitivity Analysis of Receipt of ERBB2-Targeted Therapies Through the Duration of the Study Period **eTable 7.** Sensitivity Analysis of Receipt of ERBB2-Targeted Therapies Over Time

Supplement 2.
Data Sharing Statement
