# Supplementary material for: Racial and Ethnic Disparities in Receipt of ERBB2-Targeted Therapy for Breast Cancer, 2010-2020
Source: JAMA Netw Open. 2025 May 1;8(5):e258086. doi: 10.1001/jamanetworkopen.2025.8086 (PMC12046428; doi:10.1001/jamanetworkopen.2025.8086)
Supplement: Supplement 1. — eTable 1. ERBB2-Targeted Therapies Included in Analysis eTable 2. Cohort Characteristics by Treatment Status eTable 3. Receipt of Various ERBB2-Targeted Therapies Among Medicare Beneficiaries With ERBB2-Positive Breast Cancer, by Race and Ethnicity eTable 4. Results of Multivariable Model Through the Duration of the Study Period eTable 5. Results of Multivariable Time-Specific Models eTable 6. Sensitivity Analysis of Receipt of ERBB2-Targeted Therapies Through the Duration of the Study Period eTable 7. Sensitivity Analysis of Receipt of ERBB2-Targeted Therapies Over Time [file jamanetwopen-e258086-s001.pdf]

Supplemental Online Content

Krishnamurthy S, Jazowski SA, Roberson ML et al. Racial and ethnic disparities in receipt of ERBB2-targeted therapy for breast cancer. *JAMA Netw Open*. 2025;8(5):e258086. doi:10.1001/jamanetworkopen.2025.8086

eTable 1. ERBB2-Targeted Therapies Included in Analysis

eTable 2. Cohort Characteristics by Treatment Status

eTable 3. Receipt of Various ERBB2-Targeted Therapies Among Medicare Beneficiaries With ERBB2-Positive Breast Cancer, by Race and Ethnicity

eTable 4. Results of Multivariable Model Through the Duration of the Study Period

eTable 5. Results of Multivariable Time-Specific Models

eTable 6. Sensitivity Analysis of Receipt of ERBB2-Targeted Therapies Through the Duration of the Study Period

eTable 7. Sensitivity Analysis of Receipt of ERBB2-Targeted Therapies Over Time

This supplemental material has been provided by the authors to give readers additional information about their work.

© 2025 Krishnamurthy S et al. *JAMA Network Open*.

eTable 1. ERBB2-Targeted Therapies Included in Analysis

Generic Name	Brand Name	HCPCS
Trastuzumab	Herceptin	J9355
Trastuzumab-pkrb	Herzuma	Q5113
Trastuzumab-anns	Kanjinti	Q5117
Trastuzumab-dkst	Ogivri	Q5114
Trastuzumab-dttb	Ontruzant	Q5112
Trastuzumab-qyyp	Trazimera	Q5116
Pertuzumab	Perjeta	J9306/C9292
Pertuzumab/trastuzumab/hyaluronidase-zzxf	Phesgo	J9316
Trastuzumab and hyaluronidase-oysk	Herceptin Hylecta	J9356
Ado-Trastuzumab Emtansine	Kadcyla	J9354/C9131

Abbreviations: HCPCS – Healthcare Common Procedure Classification System codes

© 2025 Krishnamurthy S et al. *JAMA Network Open*.

eTable 2. Cohort Characteristics by Treatment Status

Characteristics	Study Population (n=12,765)[a]	Treated (n=6,916)[b]	Untreated (n=5,849)
**Age at diagnosis**			
≤69	3371 (26.4)	2232 (32.3)	1139 (19.5)
70-73	2913 (22.8)	1889 (27.3)	1024 (17.5)
74-79	3213 (25.2)	1779 (25.7)	1434 (24.5)
≥80	3268 (25.6)	1016 (14.7)	2252 (38.5)
**Race and ethnicity**[c]			
Black or African American	1031 (8.1)	529 (7.7)	502 (8.6)
Hispanic	879 (6.9)	444 (6.4)	435 (7.4)
Non-Hispanic White	10855 (85.0)	5943 (85.9)	4912 (84.0)
**Low-income subsidy**[d]			
Full/partial	1948 (15.3)	895 (12.9)	1053 (18.0)
None	10817 (84.7)	6021 (87.1)	4796 (82.0)
**Census region**[e]			
Northeast	4034 (31.6)	2370 (34.3)	1664 (28.5)
Midwest	941 (7.4)	529 (7.7)	412 (7.0)
South	4411 (34.6)	1859 (26.9)	2552 (43.6)
West	3379 (26.5)	2158 (31.2)	1221 (20.9)
**Year of diagnosis**			
2010	1215 (9.5)	486 (7.0)	729 (12.5)
2011	1213 (9.5)	517 (7.5)	696 (11.9)
2012	1247 (9.8)	595 (8.6)	652 (11.2)
2013	1223 (9.6)	612 (8.9)	611 (10.5)
2014	1346 (10.5)	679 (9.8)	667 (11.4)
2015	1451 (11.4)	744 (10.8)	707 (12.1)
2016	1400 (11.0)	895 (12.9)	505 (8.6)
2017	1329 (10.4)	882 (12.8)	447 (7.6)
2018	1216 (9.5)	766 (11.1)	450 (7.7)

© 2025 Krishnamurthy S et al. *JAMA Network Open*.

2019	1125 (8.8)	740 (10.7)	385 (6.6)
**Stage at diagnosis**[f]			
Localized	8364 (65.5)	4020 (58.1)	4344 (74.3)
Regional	4401 (34.5)	2896 (41.9)	1505 (25.7)
**Hormone receptor status**			
Positive	9355 (73.3)	4832 (69.9)	4523 (77.3)
Negative	3410 (26.7)	2084 (30.1)	1326 (22.7)
**Comorbidities**[g]			
0	7490 (58.7)	3993 (57.7)	3497 (59.8)
1	2631 (20.6)	1570 (22.7)	1061 (18.1)
≥2	2644 (20.7)	1353 (19.6)	1291 (22.1)

[a] Less than 1 percent of the population was male.
[b] Patients who received ERBB2-targeted therapies in the 12 months after diagnosis were classified as treated.
[c] Race/ethnicity was defined with Research Triangle Institute (RTI) categories.
[d] Receipt of Medicare Part D low-income subsidies was measured at diagnosis.
[e] Census regions were based on beneficiaries' Surveillance, Epidemiology, and End Results registry (e.g. Massachusetts was categorized as Northeast).
[f] Localized stage describes cancer that is limited to the place where it started, with no signs of spread. Regional stage describes cancer that has spread to the nearby lymph nodes, tissues or organs.[40]
[g] Comorbidities were measured in the 12 months before diagnosis using the Klabunde modification of the Charlson comorbidity score. Comorbidities captured in this score include diagnoses of acute and history of myocardial infarction, congestive heart failure, peripheral vascular disease, cerebrovascular disease, chronic obstructive pulmonary disease, dementia, hemiplegia or paraplegia, diabetes, moderate-severe renal disease, mild liver disease, moderate-severe liver disease, peptic ulcer disease, rheumatologic disease, and AIDS.

© 2025 Krishnamurthy S et al. *JAMA Network Open*.

eTable 3. Receipt of Various ERBB2-Targeted Therapies Among Medicare Beneficiaries With ERBB2-Positive Breast Cancer, by Race and Ethnicity

Time Period and ERBB2-Targeted Therapy[a]	Race and ethnicity		
	Black or African American	Hispanic	Non-Hispanic White
**2010-2011**			
Trastuzumab	100%	100%	100%
**2012-2013**			
Trastuzumab	96.74%	98.70%	97.69%
Pertuzumab	0.00%	0.00%	0.10%
Combination[b]	3.26%	1.30%	2.22%
**2014-2015**			
Trastuzumab	71.17%	67.12%	70.38%
Pertuzumab	0.00%	1.37%	0.40%
Combination[b]	28.83%	31.51%	28.97%
Ado-Trastuzumab Emtansine	0.00%	0.00%	0.24%
**2016-2017**			
Trastuzumab	51.85%	58.27%	59.87%
Pertuzumab	0.00%	0.79%	0.13%
Combination[b]	48.15%	40.16%	39.80%
Ado-Trastuzumab Emtansine	0.00%	0.79%	0.20%
**2018-2019**			
Trastuzumab	48.70%	46.55%	50.43%
Pertuzumab	0.00%	0.00%	0.39%
Combination[b]	44.35%	49.14%	45.49%
Ado-Trastuzumab Emtansine	0.87%	0.00%	0.24%
Trastuzumab and hyaluronidase-oysk	0.87%	0.86%	0.31%

© 2025 Krishnamurthy S et al. *JAMA Network Open*.

Trastuzumab-anns	3.14%	5.22%	2.59%
Trastuzumab-qyyp	0.00%	0.00%	0.86%

[a] Time intervals were based on year of diagnosis. Receipt of clinician-administered ERBB2-targeted therapy was measured in the 12 months after diagnosis.
[b] In many cases trastuzumab and pertuzumab were given in combination on the same day or beneficiaries received some other combination of pertuzumab and a trastuzumab biosimilar

© 2025 Krishnamurthy S et al. *JAMA Network Open*.

eTable 4. Results of Multivariable Model Through the Duration of the Study Period

	Adjusted RR[a]
**Age at diagnosis**	
≤69	Ref
70-73	0.97 (0.94-1.00)
74-79	0.82 (0.79-0.85)
≥80	0.46 (0.44-0.49)
**Race and ethnicity**[b]	
Black or African American	0.93 (0.87-0.98)
Hispanic	0.88 (0.82-0.93)
Non-Hispanic White	Ref
**Census region**[c]	
Northeast	Ref
Midwest	0.93 (0.88-0.99)
South	0.70 (0.67-0.73)
West	1.03 (1.00-1.07)
**Year of diagnosis**	
2010	Ref
2011	1.10 (1.01-1.20)
2012	1.21 (1.11-1.31)
2013	1.26 (1.16-1.36)
2014	1.29 (1.19-1.39)
2015	1.30 (1.20-1.41)
2016	1.61 (1.50-1.73)
2017	1.65 (1.53-1.77)
2018	1.62 (1.50-1.74)
2019	1.66 (1.54-1.79)
**Stage at diagnosis**[d]	
Localized	Ref
Regional	1.40 (1.36-1.44)
**Hormone receptor status**	

© 2025 Krishnamurthy S et al. *JAMA Network Open*.

Positive	Ref
Negative	1.16 (1.13-1.20)
**Comorbidities**[e]	
0	Ref
1	1.11 (1.07-1.15)
≥2	1.01 (0.97-1.05)

Abbreviations: RR – Risk Ratio; Ref – Reference group.

[a] Table displays adjusted risk ratios and 95% confidence limits. Adjusted risk ratios were estimated with modified Poisson regression.

[b] Race/ethnicity was defined with Research Triangle Institute (RTI) categories.

[c] Census regions were based on beneficiaries' Surveillance, Epidemiology, and End Results registry (e.g. Massachusetts was categorized as Northeast).

[d] Localized stage describes cancer that is limited to the place where it started, with no signs of spread. Regional stage describes cancer that has spread to the nearby lymph nodes, tissues or organs.[40]

[e] Comorbidities were measured in the 12 months before diagnosis using the Klabunde modification of the Charlson comorbidity score. Comorbidities captured in this score include diagnoses of acute and history of myocardial infarction, congestive heart failure, peripheral vascular disease, cerebrovascular disease, chronic obstructive pulmonary disease, dementia, hemiplegia or paraplegia, diabetes, moderate-severe renal disease, mild liver disease, moderate-severe liver disease, peptic ulcer disease, rheumatologic disease, and AIDS.

© 2025 Krishnamurthy S et al. *JAMA Network Open*.

eTable 5. Results of Multivariable Time-Specific Models

	2010-2011 Adjusted RR[a,b]	2012-2013 Adjusted RR[a,b]	2014-2015 Adjusted RR[a,b]	2016-2017 Adjusted RR[a,b]	2018-2019 Adjusted RR[a,b]
**Age at diagnosis**					
≤69	Ref	Ref	Ref	Ref	Ref
70-73	1.02 (0.92-1.14)	0.95 (0.87-1.04)	0.95 (0.88-1.03)	0.99 (0.94-1.05)	0.97 (0.91-1.03)
74-79	0.87 (0.78-0.97)	0.79 (0.72-0.87)	0.82 (0.75-0.89)	0.84 (0.78-0.89)	0.83 (0.77-0.89)
≥80	0.42 (0.36-0.49)	0.49 (0.43-0.56)	0.38 (0.34-0.44)	0.51 (0.46-0.56)	0.52 (0.47-0.58)
**Race and ethnicity**[c]					
Black or African American	0.81 (0.68-0.97)	0.96 (0.82-1.11)	1.04 (0.92-1.18)	0.87 (0.79-0.97)	0.97 (0.87-1.08)
Hispanic	0.75 (0.62-0.92)	0.74 (0.63-0.86)	0.80 (0.68-0.93)	1.01 (0.91-1.11)	1.05 (0.95-1.16)
Non-Hispanic White	Ref	Ref	Ref	Ref	Ref
**Census region**[d]					
Northeast	Ref	Ref	Ref	Ref	Ref
Midwest	0.92 (0.79-1.07)	0.90 (0.78-1.04)	0.96 (0.86-1.08)	0.97 (0.87-1.07)	0.91 (0.80-1.03)
South	0.47 (0.41-0.54)	0.52 (0.47-0.59)	0.54 (0.49-0.60)	0.96 (0.90-1.03)	0.93 (0.86-1.00)
West	0.98 (0.89-1.08)	1.07 (0.99-1.16)	1.06 (0.98-1.14)	1.04 (0.97-1.11)	1.04 (0.97-1.11)
**Stage at diagnosis**[e]					
Localized	Ref	Ref	Ref	Ref	Ref
Regional	1.63 (1.50-1.77)	1.45 (1.35-1.56)	1.35 (1.26-1.44)	1.33 (1.27-1.40)	1.37 (1.30-1.45)
**Hormone Receptor Status**					
Positive	Ref	Ref	Ref	Ref	Ref
Negative	1.14 (1.04-1.25)	1.19 (1.10-1.28)	1.10 (1.03-1.19)	1.11 (1.05-1.17)	1.25 (1.18-1.32)
**Comorbidities**[f]					
0	Ref	Ref	Ref	Ref	Ref
1	1.15 (1.04-1.27)	1.20 (1.09-1.31)	1.23 (1.14-1.33)	0.99 (0.93-1.05)	0.95 (0.88-1.02)
≥2	0.97 (0.85-1.10)	1.05 (0.95-1.16)	1.12 (1.02-1.23)	0.89 (0.83-0.96)	0.93 (0.86-1.00)

Abbreviations: RR – Risk Ratio; Ref – Reference group.
[a] Table displays adjusted risk ratios and 95% confidence limits. Adjusted risk ratios were estimated with modified Poisson regression.

© 2025 Krishnamurthy S et al. *JAMA Network Open*.

[b] Time intervals were based on year of diagnosis. Receipt of clinician-administered ERBB2-targeted therapy was measured in the 12 months after diagnosis.
[c] Race/ethnicity was defined with Research Triangle Institute (RTI) categories.
[d] Census regions were based on beneficiaries' Surveillance, Epidemiology, and End Results registry (e.g. Massachusetts was categorized as Northeast).
[e] Localized stage describes cancer that is limited to the place where it started, with no signs of spread. Regional stage describes cancer that has spread to the nearby lymph nodes, tissues or organs.[40]
[f] Comorbidities were measured in the 12 months before diagnosis using the Klabunde modification of the Charlson comorbidity score. Comorbidities captured in this score include diagnoses of acute and history of myocardial infarction, congestive heart failure, peripheral vascular disease, cerebrovascular disease, chronic obstructive pulmonary disease, dementia, hemiplegia or paraplegia, diabetes, moderate-severe renal disease, mild liver disease, moderate-severe liver disease, peptic ulcer disease, rheumatologic disease, and AIDS.

© 2025 Krishnamurthy S et al. *JAMA Network Open*.

eTable 6. Sensitivity Analysis of Receipt of ERBB2-Targeted Therapies Through the Duration of the Study Period

Race and ethnicity	Unadjusted RR[a]	Adjusted RR[a]
Sample restricted to beneficiaries diagnosed with stage I-III cancer[b]		
Black or African American	0.93 (0.87-1.00)	0.95 (0.89-1.01)
Hispanic	1.06 (0.99-1.14)	1.08 (1.01-1.15)
Non-Hispanic White	Ref	Ref
Sample restricted to female beneficiaries[c]		
Black or African American	0.94 (0.88-1.00)	0.96 (0.90-1.02)
Hispanic	0.92 (0.86-0.99)	0.93 (0.87-0.99)
Non-Hispanic White	Ref	Ref
Model adjusted for low-income subsidy status[d]		
Black or African American	0.94 (0.88-1.00)	0.96 (0.90-1.02)
Hispanic	0.93 (0.86-0.99)	0.93 (0.87-0.99)
Non-Hispanic White	Ref	Ref

Abbreviations: Ref – Reference group.

© 2025 Krishnamurthy S et al. *JAMA Network Open*.

[a] Table displays unadjusted and adjusted risk ratios and 95% confidence limits. Unadjusted and adjusted risk ratios were estimated with modified Poisson regression models.
[b] Given that the TNM staging system guides clinical practice and treatment guidelines, the sample was restricted to beneficiaries diagnosed with stage I-III ERBB2-positive breast cancer (n=7,525). Stage was defined using derived American Joint Committee on Cancer staging codes, combined staging codes, and derived extend of disease staging codes – all of which are based on the TNM system.
[c] The sample was restricted to female beneficiaries (n=12,659).
[d] Consistent with the National Academy of Medicine's (formerly Institute of Medicine) definition of racial/ethnic disparities, our primary models only adjusted for health status and geographic region. In this sensitivity analysis, we adjusted for socioeconomic status (Medicare Part D low-income subsidy status) to determine if observed racial/ethnic disparities in receipt of ERBB2-targeted therapies were attenuated.

© 2025 Krishnamurthy S et al. *JAMA Network Open*.

eTable 7. Sensitivity Analysis of Receipt of ERBB2-Targeted Therapies Over Time

Race and ethnicity	2010-2011 Adjusted RR[a,b]	2012-2013 Adjusted RR[a,b]	2014-2015 Adjusted RR[a,b]	2016-2017 Adjusted RR[a,b]	2018-2019 Adjusted RR[a,b]
Sample restricted to beneficiaries diagnosed with stage I-III cancer[c]					
Black or African American	0.85 (0.70-1.03)	0.96 (0.83-1.10)	1.06 (0.94-1.19)	0.91 (0.80-1.03)	0.96 (0.83-1.11)
Hispanic	1.06 (0.85-1.31)	1.03 (0.87-1.22)	1.10 (0.96-1.27)	1.10 (0.97-1.24)	1.10 (0.97-1.26)
Non-Hispanic White	Ref	Ref	Ref	Ref	Ref
Sample restricted to female beneficiaries[d]					
Black or African American	0.82 (0.69-0.98)	1.00 (0.86-1.16)	1.07 (0.95-1.21)	0.90 (0.81-1.00)	1.01 (0.91-1.13)
Hispanic	0.82 (0.66-1.01)	0.81 (0.69-0.95)	0.83 (0.71-0.97)	1.04 (0.94-1.15)	1.11 (1.00-1.23)
Non-Hispanic White	Ref	Ref	Ref	Ref	Ref
Model adjusted for low-income subsidy status[e]					
Black or African American	0.84 (0.71-1.00)	1.00 (0.86-1.16)	1.07 (0.95-1.22)	0.89 (0.80-0.99)	1.01 (0.91-1.13)
Hispanic	0.82 (0.66-1.01)	0.80 (0.68-0.94)	0.84 (0.72-0.98)	1.03 (0.94-1.14)	1.11 (1.00-1.23)
Non-Hispanic White	Ref	Ref	Ref	Ref	Ref

Abbreviations: RR – Risk ratio; Ref – Reference group.
[a] Table displays adjusted risk ratios and 95% confidence limits. Adjusted risk ratios were estimated with modified Poisson regression.
[b] Time intervals were based on year of diagnosis. Receipt of clinician-administered ERBB2-targeted therapy was measured in the 12 months after diagnosis.

© 2025 Krishnamurthy S et al. *JAMA Network Open*.

[b] Given that the TNM staging system guides clinical practice and treatment guidelines, the sample was restricted to beneficiaries diagnosed with stage I-III ERBB2-positive breast cancer (n=7,525). Stage was defined using derived American Joint Committee on Cancer staging codes, combined staging codes, and derived extend of disease staging codes – all of which are based on the TNM system.
[c] The sample was restricted to female beneficiaries (n=12,659).
[d] Consistent with the National Academy of Medicine’s (formerly Institute of Medicine) definition of racial/ethnic disparities, our primary models only adjusted for health status and geographic region. In this sensitivity analysis, we adjusted for socioeconomic status (Medicare Part D low-income subsidy status) to determine if observed racial/ethnic disparities in receipt of ERBB2-targeted therapies were attenuated.

© 2025 Krishnamurthy S et al. *JAMA Network Open*.
