# Supplementary material for: Racial and Ethnic Disparities in Receipt of ERBB2-Targeted Therapy for Breast Cancer, 2010-2020
Source: JAMA Netw Open. 2025 May 1;8(5):e258086. doi: 10.1001/jamanetworkopen.2025.8086 (PMC12046428; doi:10.1001/jamanetworkopen.2025.8086)
Supplement: Supplement 2. — Data Sharing Statement [file jamanetwopen-e258086-s002.pdf]

Data Sharing Statement

Krishnamurthy. Racial and Ethnic Disparities in Receipt of ERBB2-Targeted Therapy for Breast Cancer, 2010-2020. *JAMA Netw Open*. Published May 01, 2025.
doi:10.1001/jamanetworkopen.2025.8086

Data
Data available: No

Additional Information
Explanation for why data not available: The SEER-Medicare linked data underlying this study cannot be shared due to provisions outlined in the data use agreement between the study team and the National Cancer Institute (NCI). Researchers interested in obtaining these data can submit a project-specific data request to the NCI.
